# A Manual of Materia Medica and Therapeutics, Including the Preparations of the Pharmacopœias of London, Edinburgh, and Dublin, with Many New Medicines

**Published:** 1847-01

**Authors:** 


Art. VII.—*A Manual of Materia Medica and Therapeutics, including the preparations of the Pharmacopœias of London, Edinburgh, and Dublin, with many new Medicines.* By J. Forbes Royle, m.d. f.r.s., Professor of Materia Medica and Therapeutics, King's College, London. With ninety-eight woodcuts.—*London,* 1847. 8vo, pp. 716.

This is another member of that beautiful and cheap series of Manuals published by Mr. Churchill for the benefit of the student and general practitioner. In the execution of the woodcuts, particularly of the plants, flowers, and fruits, Mr. Bagg seems almost to have exceeded his former doings, while the paper-maker and printer have performed their parts with equal credit. In regard to the yet more essential constituent, the literary portion of the work, no one who is acquainted with the former productions of Dr. Royle will doubt that the author has discharged his duties with the same skill as the artist. The work is, indeed, a most valuable one, and will fill up an important gap that existed between Dr. Pereira's most learned and complete system of materia medica, and the class of productions at the other extreme, which are necessarily imperfect from their small extent. Such a work as this does not admit of analysis and scarcely of detailed critical examination. It would, however, be injustice to the learned author not to state that, in addition to what former works on the subject necessarily contained, the reader will find here not a little that is either original, or introduced for the first time, more especially in the details of botany and natural history, and in what may be termed the archæology of drugs.

